# Controllable Valley Polarization and Strain Modulation in 2D 2H–VS_2_/CuInP_2_Se_6_ Heterostructures

**DOI:** 10.3390/nano12142461

**Published:** 2022-07-18

**Authors:** Fan Yang, Jing Shang, Liangzhi Kou, Chun Li, Zichen Deng

**Affiliations:** 1 School of Mechanics, Civil Engineering and Architecture, Northwestern Polytechnical University, Xi’an 710072, China; fanyang@mail.nwpu.edu.cn (F.Y.); lichun@nwpu.edu.cn (C.L.); 2 School of Materials Science and Engineering, Shaanxi University of Science and Technology, Xi’an 710021, China; 3 School of Mechanical, Medical and Process Engineering, Queensland University of Technology, Brisbane, QLD 4000, Australia; liangzhi.kou@qut.edu.au; 4 Research and Development Institute of Northwestern Polytechnical University in Shenzhen, Shenzhen 518057, China

**Keywords:** valley polarization, strain modulation, 2H–VS_2_/CIPSe heterostructures, first–principles calculations

## Abstract

Two–dimensional (2D) transition metal dichalcogenides endow individually addressable valleys in momentum space at the K and K’ points in the first Brillouin zone due to the breaking of inversion symmetry and the effect of spin–orbit coupling. However, the application of 2H–VS_2_ monolayer in valleytronics is limited due to the valence band maximum (VBM) located at the Γ point. Here, by involving the 2D ferroelectric (FE) CuInP_2_Se_6_ (CIPSe), the ferrovalley polarization, electronic structure, and magnetic properties of 2D 2H–VS_2_/CIPSe heterostructures with different stacking patterns and FE polarizations have been investigated by using first–principles calculations. It is found that, for the energetically favorable AB–stacking pattern, the valley polarization is preserved when the FE polarization of CIPSe is upwards (CIPSe↑) or downwards (CIPSe↓) with the splitting energies slightly larger or smaller compared with that of the pure 2H–VS_2_. It is intriguing that, for the FE CIPSe↑ case, the VBM is expected to pass through the Fermi energy level, which can be eventually achieved by applying biaxial strain and thus the valleytronic nature is turned off; however, for the CIPSe↓ situation, the heterostructure basically remains semiconducting even under biaxial strains. Therefore, with the influence of proper strains, the FE polar reversal of CIPSe can be used as a switchable on/off to regulate the valley polarization in VS_2_. These results not only demonstrate that 2H–VS_2_/CIPSe heterostructures are promising potential candidates in valleytronics, but also shed some light on developing practical applications of valleytronic technology.

## 1. Introduction

With the rapid development of two–dimensional (2D) materials [1,2,3,4,5,6], graphene and graphene–like single–layer materials as well as their heterostructures have aroused widespread concern because of their unique electric [7,8,9,10,11], magnetic [12,13,14,15,16,17,18], optical [19,20,21,22], and mechanical properties [23,24,25]. In particular, as promising materials in valleytronics where the valley state is the extreme point of energy dispersion located at K and K’ in the first Brillouin zone [26,27], 2D transition metal dichalcogenides (TMDs) have recently attracted considerable attention since they are both physically intriguing and practically appealing to be used in nanoscale optoelectronic devices [28,29,30,31].

It is known that, for the monolayer Group–V TMDs, such as VX_2_ (X = S, Se, Te), the coexistence of the spin–orbit coupling (SOC) effect and exchange interaction of localized *d* electrons result in spontaneous valley polarization [32]. The monolayer VS_2_ has been confirmed to have an intrinsic ferromagnetic semiconducting property, which could be a suitable candidate for constructing 2D magnetic Van der Waals (vdW) heterostructures [33]. The VS_2_ monolayer exhibits magnetic characteristics with V atoms contributing magnetism (~1 μB). Similar to MoS_2_, it has three stable structures (2H, 1T, and 1T’), among which the 2H structure is semiconducting and 1T and 1T’ are metallic. 2H–VS_2_ exhibits the spontaneous valley polarization due to the intrinsic exchange interaction between V 3*d* electrons, which is equivalent to a permanent magnetic field. Thus, the concept of the ferrovalley material has been proposed in monolayer VS_2_. The various properties of pure VS_2_ have been studied since 2013 in both theory and experiment [34,35,36], mainly focusing on the energy band structure and the magnetic property of the material. Since 2017, VS_2_ has formed heterojunctions with graphene, Mxene, CN materials, and TMDs to obtain the regulation of electronic structure and the potential applications in lithium (sodium/zinc) ion batteries [37,38,39,40].

Due to the bistable and nonvolatile characteristics of ferroelectric (FE) materials, it is desirable to combine VS_2_ with a suitable FE material to improve its controllability in valleytronic structures. In this work, a newly discovered 2D Van der Waals (vdW) FE CuInP_2_Se_6_ (CIPSe) with switchable polarization directions along the out–of–plane direction is selected to construct a heterostructure with VS_2_, which has not been investigated previously. As room–temperature ferroelectrics, CIPSe possesses the atomic structure containing a sulfur framework with the octahedral voids filled by the Cu, In, and P–P triangular patterns. Bulk crystals are composed of vertically stacked, weakly interacting layers packed by vdW interactions. Owing to the site exchange between Cu and P–P pair from one layer to another, a complete unit cell consists of two adjacent monolayers to fully describe the material’s symmetry. 

In order to investigate whether valley and spin splitting can be modulated in 2H–VS_2_ combined with a FE CIPSe layer, and how FE polarization affects the electronic structure of 2H–VS_2_, considering the small lattice mismatch of 1.18%, the 2H–VS_2_/CIPSe heterostructures are established in this work. As a result, for the energetically favorable stacking pattern (AB–stacking) and the most stable magnetic ground state (ferromagnetic phase), valley splitting energies of the combined system could be adjusted by the orientation of FE polarization in CIPSe. It is further shown that the applied biaxial strain can effectively modulate the valley polarization of the system, which suggests that 2H–VS_2_/CIPSe heterostructures are the potential candidates in valleytronics.

## 2. Method and Technique Details

The first–principles calculations based on the density–functional theory (DFT) have been performed on all structures by using the Vienna Ab initio simulation (VASP) package [41], implemented within the generalized gradient approximation (GGA) and Perdew–Burke–Ernzerhof (PBE) function [42] for the calculation of geometries and the electronic structures. The SOC effect was considered in all calculations. To describe the on–site Coulomb interaction, the Hubbard parameters *U* and *J* are taken as 3 eV and 1 eV for the transition metal V atom according to the previously reported value [43]. In the present simulations, the total energy and residual force were convergent to an accuracy of 10^−4^ eV and 10^−2^ eV/Å, respectively, by using a plane–wave energy cutoff of 450 eV. The Brillouin zone was represented by a Monkhorst–Pack special *k*–point meshes of 7 × 7 × 1 for all structures. The heterostructures are composed of CIPSe unit cell and 2 × 2 × 1 VS_2_ supercell along the vertical direction. The in–plane lattice parameters of monolayers CIPSe and 2H–VS_2_ are 6.454 and 6.378 Å, respectively, with lattice mismatch of 1.18%. A 25 Å–thick vacuum layer was added to avoid the interaction between adjacent layers. The vdW correction with zero damping DFT–D3 method of Grimme [44] was used to describe the interlayer interactions between VS_2_ and CIPSe layers.

## 3. Results and Discussion 

### 3.1. Electronic Structures of Pristine 2H–VS_2_

**Figure 1:**
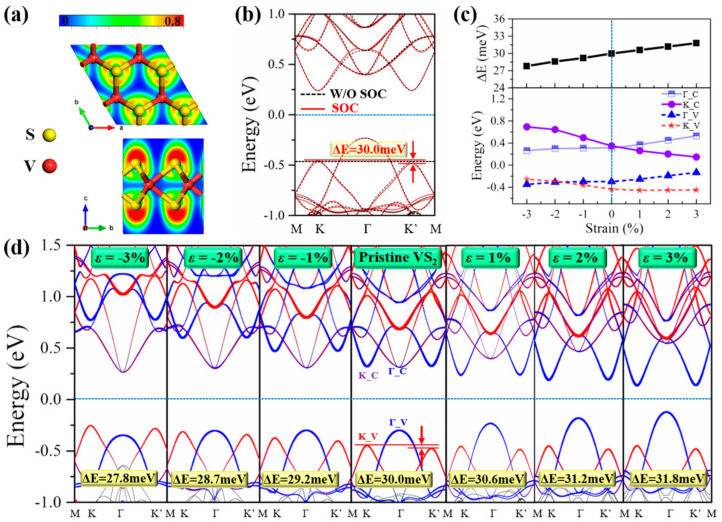
Valley splitting and band structures of pristine 2H–VS_2_. (**a**) Electron Localization Function (ELF) map of pristine 2H–VS_2_ with top and front views of the optimized 2H–VS_2_; (**b**) band structures without SOC (black dashed curves) and with SOC (red solid curves) of pristine 2H–VS_2_; (**c**) valley splitting energies (ΔE) (upper panel) and conduction/valence band energies at K and G positions (lower panel) of VS_2_ under biaxial strains; (**d**) valley splitting and band structures with SOC of VS_2_ under biaxial strains. The red and blue lines denote the contributions from V–$d_{xy}+d_{x^{2}-y^{2}}$ and V–$d_{z^{2}}$ orbitals, respectively. The Fermi level is set to zero.

Before investigating the 2H–VS_2_/CIPSe heterostructures, the valley splitting and band structures of pristine 2H–VS_2_ are studied firstly. The top and front views of the optimized pure 2H–VS_2_ structure with its electron localization function (ELF) map are shown in Figure 1a, where the red area (valued 0.8) and blue area (values 0) represent higher and lower electron localizations, respectively. It is shown that the electrons in 2H–VS_2_ are highly localized in the areas close to the S atoms. Note that there exist two configurations of VS_2_, namely, 1T and 2H phases. As shown in Figure S1 of the Supplementary Materials, based on the 2H phase, the S atoms in the upper and lower layers move the same distance along the opposite direction in the plane to form the 1T phase. The spin–resolved energy band structures shown in Figure S1 indicate that 1T–VS_2_ is a conductor; thus, the semiconducting 2H–VS_2_ structure is mainly concentrated in the present work. Just as the calculated band structures of pristine 2H–VS_2_ shown in Figure 1b, besides the semiconducting feature, the valley splitting only appears when the SOC is considered (red solid curves), since the energies at points K and K’ are equivalent without considering the SOC (black dashed curves). The magnetocrystalline anisotropy energy (MAE) is defined as the energy difference between the perpendicular and parallel magnetization directions by the force theorem, namely *MAE* = *E*[1] − *E*[100], where *E*[1] and *E*[100] represent the total energies of out–of–plane and in–plane spin alignments, respectively. Positive and negative MAE indicate in–plane and out–of–plane MA, respectively. The calculated MAE value is −0.158 meV/unit_cell for the pristine VS_2_ monolayer, suggesting that the easy axis of magnetization is along the out–of–plane direction and the VS_2_ monolayer material has obvious magnetic anisotropy. This is consistent with the results of previous reports [45,46].

Figure 1c shows the variation of the energies of the conduction band minimum (CBM) and the valence band maximum (VBM) at high symmetry points K/G in the Brillouin region according to the calculated band structures with SOC of VS_2_ under biaxial strains as shown in Figure 1d. It can be seen that the VS_2_ monolayer has an intrinsic valley splitting of 30 meV. Note that, for the pristine VS_2_, the obtained valley splitting energies (ΔE) of the strained structures are only slightly larger (stretching) or smaller (compressing) compared with the unstrained structure. This trend of strain tuned ΔE is consistent with the results in previous study [33]. The result indicates that the valley splitting energy of VS_2_ can be regulated by the strain, but the variation range is small and the effect is relatively weak. Therefore, it is necessary to seek other diversified modulation methods. For instance, constructing a heterostructure with a FE substrate is an effective way, since the FE polarization possesses bistability of polarization upward (P↑) and polarization downward (P↓), and, generally, the two states can be reversibly switched with the influence of an external field (such as electric field or force field). As mentioned previously, in the present work, the CIPSe is chosen as the FE substrate, and the 2H–VS_2_/CIPSe heterostructures are established to further explore the physical mechanism for the control of the valley polarization of the structure by use of the FE polarization as well as the strain.

### 3.2. Structural and Electronic Properties of 2H–VS_2_/CIPSe Heterostructures

In the present work, according to the three cases that Cu atom in CIPSe is facing S atom (S for AA–stacking), hexagonal center (H for AB–stacking), and V atom (V for AC–stacking) in 2H–VS_2_, three different stacking patterns are considered to investigate the electronic structure and magnetic properties of 2D 2H–VS_2_/CIPSe heterostructures (see Figure S2 in the Supplementary Materials). The calculated total energies for each stacking pattern with respect to different polarization directions are listed in Table 1, in which both ferromagnetic (FM) and antiferromagnetic (AFM) ground states are considered. It can be seen from Table 1 that ferromagnetic 2H phases are more stable, and will be further studied in the subsequent calculations.

**Figure 2:**
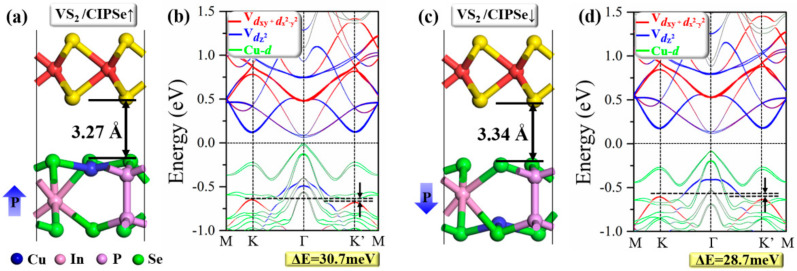
Atomic structures with interlayer distance and calculated band structures with SOC of (**a**,**b**) 2H–VS_2_/CIPSe↑ and (**c**,**d**) 2H–VS_2_/CIPSe↓ heterostructures. The arrows “↑” and “↓” represent ferroelectric upward and downward polarizations. The solid lines in red, blue, and green represent the contributions from V–$d_{xy}+d_{x^{2}-y^{2}}$, V–$d_{z^{2}}$ and Cu–*d* orbitals, respectively. The Fermi level is set to zero.

The optimized atomic structures with interlayer distance and calculated band structures of 2H–VS_2_/CIPSe heterostructures considering SOC with opposite directions of FE polarizations are shown in Figure 2. Compared with the intrinsic valley splitting of the pristine VS_2_ monolayer shown in Figure 1b, the valley polarization is preserved; when the FE polarization of CIPSe is pointing upwards (2H–VS_2_/CIPSe↑) or downwards (2H–VS_2_/CIPSe↓), the 2H–VS_2_ layer with the splitting energies (ΔE) is slightly larger (30.7 meV) or smaller (28.7 meV) than that of the pure 2H–VS_2_ (30 meV). Notably, the MAE in VS_2_/CIPSe with upward (↑) and downward FE polarizations (↓) are 0.191 and 0.187 meV/unit_cell, respectively. The positive values indicate the easy axis of magnetization is parallel to the 2D plane. The in–plane magnetization was also found in the 2H–VTe_2_ monolayer [47]. Similar to the MAE of the pristine VS_2_ monolayer, the MAE in VS_2_/CIPSe provides the precondition to discuss the ferrovalley. Furthermore, from the band structures shown in Figure 2b,d, for both situations of CIPSe↑ and CIPSe↓, the CBM of the heterostructures are mainly contributed by V $d_{z^{2}}$, $d_{xy}$, and $d_{x^{2}-y^{2}}$ orbitals, while the VBM is mainly contributed by Cu *d* orbitals. The degenerate V $d_{xy}$ and $d_{x^{2}-y^{2}}$ orbitals in the pristine monolayer VS_2_ are lifted in the CIPSe↑ and CIPSe↓ models, which can be attributed to the influence of interfacial interaction. In particular, the VBM in the VS_2_/CIPSe↑ case is almost passed through the Fermi energy level (Figure 2b), which is expected to be achieved by applying biaxial strain and thus the valleytronic nature can be turned off. At the same time, sufficient large valley splitting energy in VS_2_/CIPSe heterostructures ensures that valley behavior can be maintained at room temperature. The magnetic behavior in the ferromagnetic VS_2_ monolayer can be modulated in the VS_2_/CIPSe model by reversing the direction of FE polarizations of CIPSe, achieving the magnetoelectric coupling between VS_2_ and CIPSe.

**Figure 3:**
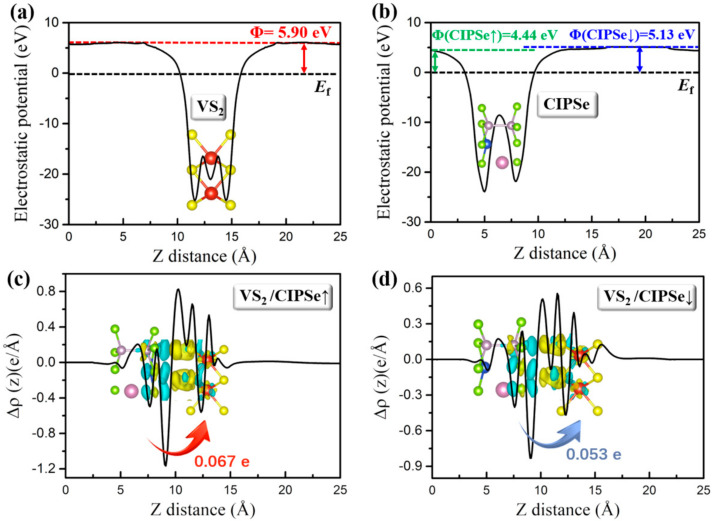
(**a,b**) Electrostatic potential and work functions (Φ) for pristine 2H–VS_2_ (**a**) and CIPSe (**b**), respectively. The electrostatic potential is defined with respect to Fermi energy (EF). Φ therefore indicates the required energy to remove an electron at Fermi level from the material to a state at rest in the vacuum nearby the surface. (**c**,**d**) The plane–integrated electron density difference along the vertical direction for the VS_2_/CIPSe↑(**c**) and VS_2_/CIPSe↓ (**d**) heterojunctions. The insets represent the 3D isosurface of differential charge densities for the VS_2_/CIPSe heterojunctions. The yellow and cyan regions represent electron accumulation and depletion, respectively, and the isosurface value is set to be 0.0002 e/Å^3^.

To precisely confirm the charge transfer between the layers of 2H–VS_2_/CIPSe heterostructures, the electrostatic potential and work functions (Φ) for pristine 2H–VS_2_ and CIPSe are calculated, respectively. The work function can be defined as the minimum energy required moving an electron from the interior of a solid to the surface of the object. The smaller the energy required for an electron at the surface of the Fermi energy to escape to the vacuum energy level, the smaller the value of the work function will be. As shown in Figure 3a,b, the calculated work functions of pristine 2H–VS_2_, CIPSe↑, and CIPSe↓ monolayers are 5.90, 4.44, and 5.13 eV, respectively, indicating that the electrons flow from the CIPSe layer to the 2H–VS_2_ layer when the 2H–VS_2_ monolayer combines with the CIPSe monolayer to form a heterostructure due to the smaller work function of CIPSe. Eventually, the 2H–VS_2_ layer gains negative electrons while the CIPSe layer accumulates positive charge. Since the work function of CIPSe↑ (4.44 eV) is smaller than that of the CIPSe↓ (5.13 eV), it is easier to lose electrons for the CIPSe↑ case when FE polarization is upward, which is consistent with the result of Bader charge. Specifically, for the VS_2_/CIPSe↑, the charge with an amount of 0.067 electrons is transferred from CIPSe to VS_2_; meanwhile, for the case of VS2/CIPSe↓, the charge transfer occurs the other way around with the value of 0.053 electrons, as demonstrated by the plane–integrated electron density difference along the vertical direction shown in Figure 3c,d. In addition, this result is also consistent with the optimized interlayer distance for each situation. As shown in Figure 2, the interlayer distance of VS_2_/CIPSe↑ (3.27 Å) is smaller than the corresponding value of VS_2_/CIPSe↓, which results in stronger electron interactions between the layers. It should be noted that the present results also indicate different strain effects that will be further analyzed in the following section.

### 3.3. In-Plain Biaxial Strain Effect on the 2H–VS_2_/CIPSe Heterostructures

**Figure 4:**
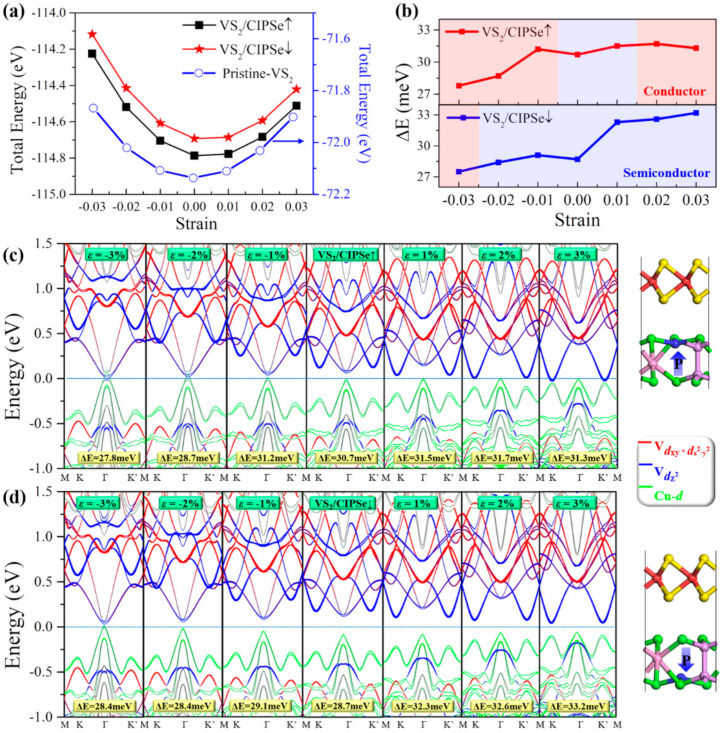
In–plain biaxial strain effect on valley splitting and band structures with SOC of VS_2_/CIPSe heterostructures. (**a**) relationships between total energies and biaxial strains (black, red, and blue curves are for VS_2_/CIPSe↑, VS_2_/CIPSe↓ and pristine VS_2_, respectively); (**b**) valley splitting energies (ΔE) under biaxial strains for VS_2_/CIPSe↑, VS_2_/CIPSe↓, red and blue area indicate conductor and semiconductor behaviors. (**c**,**d**) valley splitting and band structures with SOC of VS_2_/CIPSe heterostructures under biaxial strains with upward (**c**) and downward (**d**) ferroelectric polarization. The red, blue, and green lines denote the contributions from V–$d_{xy}+d_{x^{2}-y^{2}}$, V–$d_{z^{2}}$ and Cu–*d* orbitals, respectively. The Fermi level is set to zero.

It is well–known that strain engineering is an effective method to modulate the electronic and transport characteristics in 2D nanoscale electronic devices. In practical applications, strain engineering can achieve different compressive and tensile strains by bending the substrate attached to 2D materials [33,48]. Due to the mismatch of lattice constants between VS_2_ and CIPSe, it is necessary to evaluate the most stable lattice constant of the heterostructure. Figure 4a shows the relationship between the total energies and the applied biaxial strains for the pure VS_2_, 2H–VS_2_/CIPSe↑, and 2H–VS_2_/CIPSe↓ structures, respectively. For each system, the minimum total energy appears in the unstrained case, which is reasonable and confirms the fully relaxed structures as well.

Figure 4b shows the valley splitting energies (ΔE) of the VS_2_/CIPSe↑ and VS_2_/CIPSe↓ with respect to the applied biaxial strains. Similar to the result shown in Figure 2c, ΔE of the strained structures are only slightly fluctuated compared with that of the unstrained structure. Nevertheless, for the VS_2_/CIPSe↑ system, under the influence of biaxial strain, both stretching and compressing strains result in the transform from semiconductor to conductor. Specifically, when the heterostructure is stretched, the conducting feature mainly originates from the fact that the CBM at points K and K’ cross the Fermi level; meanwhile, when the system is compressed, both the CBM and VBM at the central high–symmetry point G pass through the Fermi level, which also leads to a conducting characteristic of the heterostructure. Since the valleytronic polarization is only effective in a semiconductor, the above situation can be considered as the disappearance of valleytronic characteristics in the material. In fact, a similar situation was also reported in 2H–VSe_2_/P↑ heterostructure in recent literature [49], in which the half–metallic characteristic of the system indicates that the valleytronic nature is turned off. On the other hand, the VS_2_/CIPSe↓ heterostructure remains semiconducting with the influence of most strains except for the situation of the −0.03 strain. The corresponding partial density of states (PDOS) for both spin–up and spin–down electrons under biaxial strains for AB–stacking VS_2_/CIPSe↑, pristine VS_2_, and VS_2_/CIPSe↓heterostructures are shown in Figure S3 of the Supplementary Materials. In addition, for the intralayer FM configuration of VS_2_, the total magnetic moment of pristine VS_2_ remains unchanged, as seen with the black line in Figure S3d. In contrast, the magnetic moments of VS_2_/CIPSe heterostructures increase with the applied tensile strain and decline with the compressive strains. Particularly, the magnetic moment of VS_2_/CIPSe↑is more sensitive to the external strains, compared to VS_2_/CIPSe↓ systems, due to the stronger interface interaction between VS_2_ and CIPSe↑, as the differential charge density shown in Figure 3c,d. It should be noted that, in DFT calculations, the temperature is considered as 0 K, and it is true that the valley splitting energy changes slightly, which is hard to be detected in experiments at room temperature. However, when combining the electronic structures under biaxial strains, the transition from semiconductor to metal helps to turn on/off the valley polarizations. This is intriguing and important as it demonstrates that the biaxial strain combined with the controllable FE polarizations could serve as a switch for the valleytronic nature of the material.

**Figure 5:**
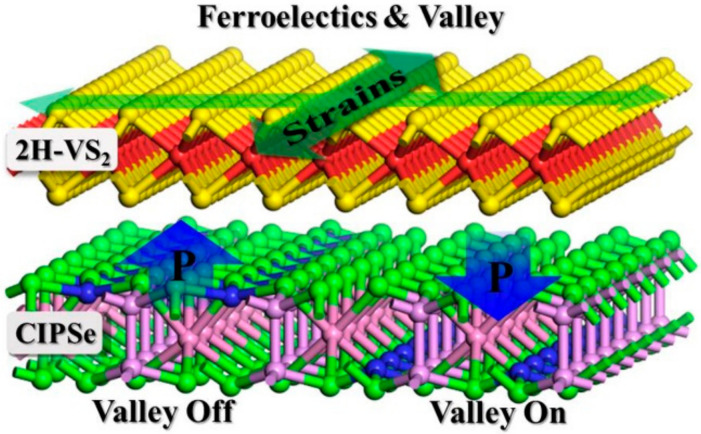
The schematic design for applications of the tunable ferrovalley of 2H–VS_2_ monolayer under both in–plane strains and ferroelectric CIPSe substrate in 2H–VS_2_/CIPSe heterostructures.

As detailed above, the valley splitting energy of pure 2H–VS_2_ monolayer can be tuned by external strains within a quite small range from 27.8 to 31.8 meV. Therefore, a ferroelectric substrate of CIPSe monolayer is added to form heterostructures. By utilizing the different interlayer effects between 2H–VS_2_ and CIPSe layers with upwards and downwards FE polarizations, respectively, a ferrovalley switch has been achieved when in–plain strains are applied simultaneously. Accordingly, we can design an ideal switchable valleytronic device based on 2H–VS_2_/CIPSe heterostructures, as the schematic design plotted in Figure 5. Such a scheme for designing valleytronic switch in 2H–VS_2_/CIPSe heterostructures is also expected to be applicable for other intrinsic valley TMDs combined with FE substrates.

## 4. Conclusions

In this paper, the ferrovalley polarization, electronic structure, and magnetic properties of 2D 2H–VS_2_/CIPSe heterostructures with different stacking patterns and FE polarization have been investigated by using first–principles calculations. It is shown that the valley splitting of monolayer VS_2_ can be affected by the CIPSe substrate with FE polarization. In addition, the reversed orientation of FE polarization of the CISPe substrate can modulate the magnitude of valley splitting. It is found that the biaxial strains play an important role in the modulation of valleytronic nature of the heterostructures. Although the valley splitting energies of the strained structures are only slightly fluctuated compared with that of the unstrained structure, the biaxial strain combined with the FE polarization could serve as an effective switch for the valleytronic nature of the material. The controllable valley polarization and the strain modulation mechanism revealed in the present work demonstrate that the 2H–VS_2_/CIPSe heterostructures are promising candidates for the applications in 2D valleytronic devices.

## Figures and Tables

**Table 1:** Calculated total energies (eV) of 2H–VS_2_/CIPSe heterostructures for each stacking pattern with respect to different polarization directions and magnetic ground states.

Polarization Direction	Magnetic Ground State	AA–Stacking (S)	AB–Stacking (H)	AC–Stacking (V)
CIPSe↑	FM	−114.666 eV	−114.751 eV	−114.727 eV
CIPSe↓	FM	−114.618 eV	−114.654 eV	−114.617 eV
CIPSe↑	AFM	−114.193 eV	−114.287 eV	−114.256 eV
CIPSe↓	AFM	−114.137 eV	−114.180 eV	−114.136 eV

## Supplementary Materials

The following supporting information can be downloaded at: https://www.mdpi.com/article/10.3390/nano12142461/s1. 1T and 2H configurations of VS_2_ and the corresponding spin–resolved energy band structures; three kinds of stacking patterns of 2H–VS_2_/CIPSe heterostructures; the PDOS and total magnetic moments of the AB–stacking heterostructures and the pristine VS_2_ under biaxial strains.
