# Supplementary material for: Controllable Valley Polarization and Strain Modulation in 2D 2H–VS2/CuInP2Se6 Heterostructures
Source: Nanomaterials (Basel). 2022 Jul 18;12(14):2461. doi: 10.3390/nano12142461 (PMC9315968; doi:10.3390/nano12142461)
Supplement: Supplementary file 1 [file nanomaterials-12-02461-s001.zip › nanomaterials-1797759-supplementary.pdf]

Supplementary Materials:

Controllable Valley Polarization and Strain Modulation in 2D 2H–VS_2/$CuInP_2Se_6$ Heterostructures

Fan Yang[1], Jing Shang[2*], Liangzhi Kou[3], Chun Li[1,4], Zichen Deng[1*]

[1]*School of Mechanics, Civil Engineering and Architecture, Northwestern Polytechnical University, Xi'an 710072, China*

[2]*School of Materials Science & Engineering, Shaanxi University of Science & Technology, Xi'an 710021, China*

[3]*School of Mechanical, Medical and Process Engineering, Queensland University of Technology, Brisbane, Queensland 4000, Australia*

[4]*Research & Development Institute of Northwestern Polytechnical University in Shenzhen, 518057, China*

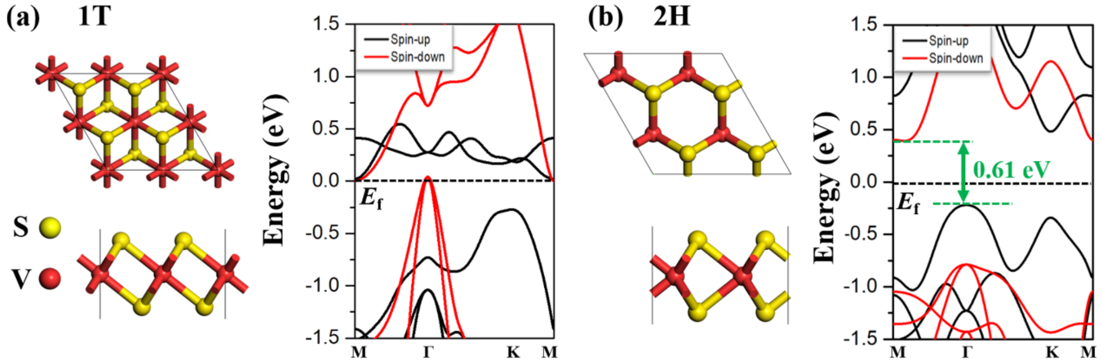

Figure S1 (a) 1T and (b) 2H configurations of VS_2 and the corresponding spin–resolved energy band structures.

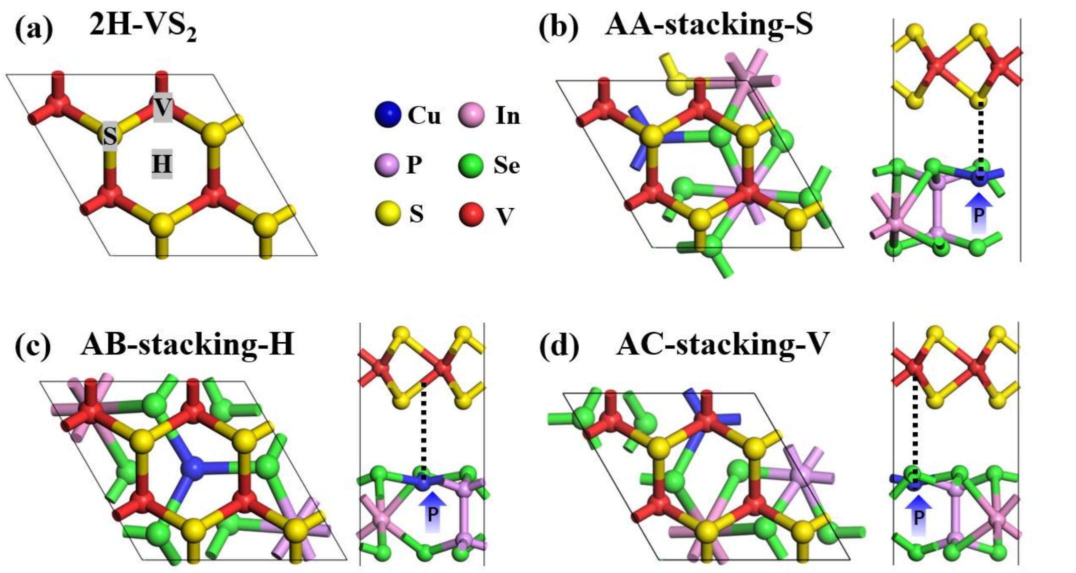

Figure S2 Three kinds of stacking patterns. (a) The stacking positions for S, H, and V, (b) AA–stacking, (c) AB–stacking, and (d) AC–stacking of 2H–VS_2/CIPSe heterostructures.

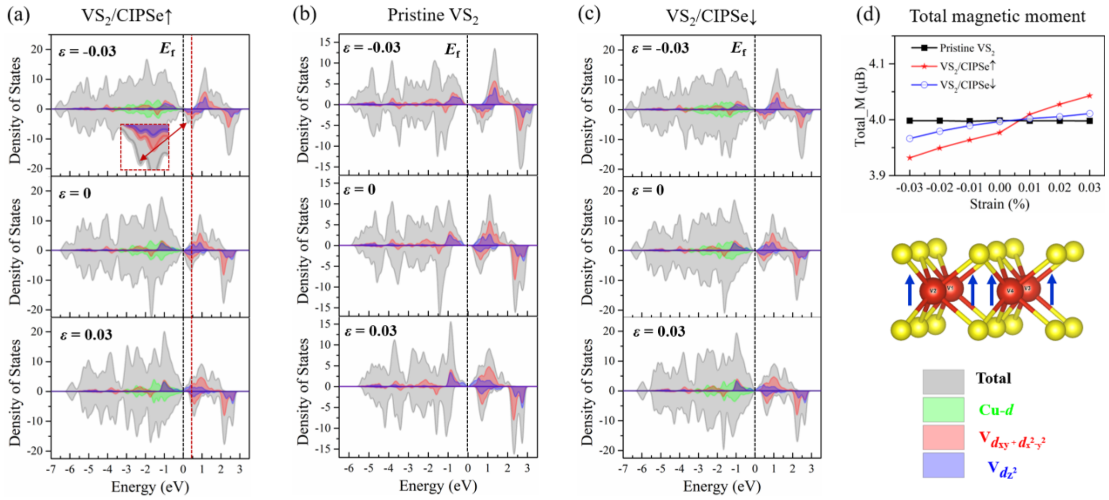

Figrue S3 The partial density of states (PDOS) for both spin–up and spin–down electrons under biaxial strain for (a) AB–stacking VS_2/CIPSe↑, (b) pristine VS_2, and (c) AB–stacking VS_2/CIPSe↓. (d) The total magnetic moments of VS_2/CIPSe↑, pristine VS_2, and VS_2/CIPSe↓ under biaxial strains, respectively.
